# Supplementary material for: AAV-mediated expression of a new conformational anti-aggregated α-synuclein antibody prolongs survival in a genetic model of α-synucleinopathies
Source: NPJ Parkinsons Dis. 2023 Jun 15;9:91. doi: 10.1038/s41531-023-00542-9 (PMC10272115; doi:10.1038/s41531-023-00542-9)
Supplement: Supplementary file 1 — Supplemental Material [file 41531_2023_542_MOESM1_ESM.pdf]

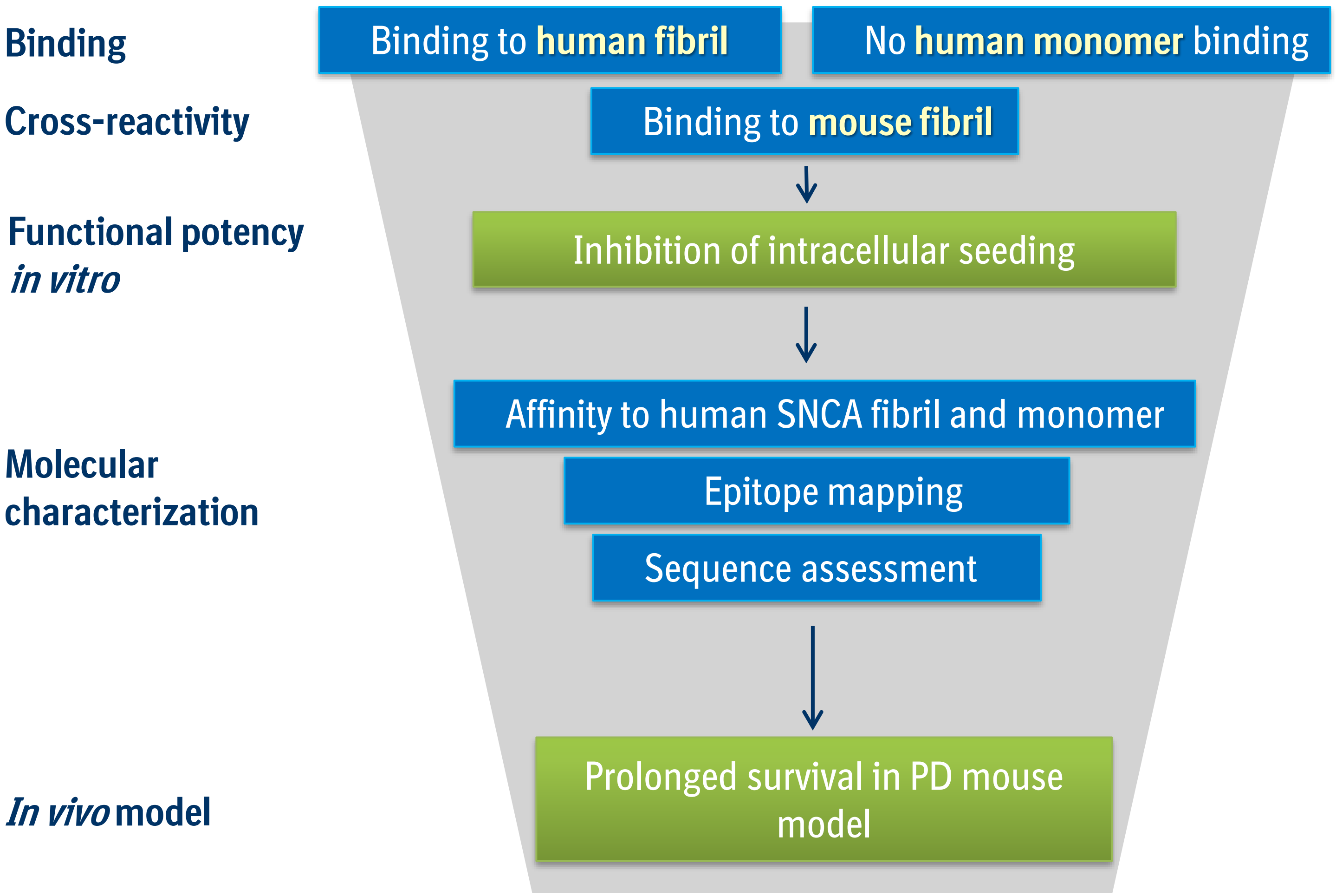

Supplementary Figure 1. Screening cascade employed to identify fibril-specific anti-α-synuclein antibodies.

Hybridoma supernatants were screened by ELISA for binding activity against fibrillar human and mouse α-synuclein. Supernatants of hybridomas negative for binding towards monomeric human α-synuclein were tested in a cellular assay assessing their capacity to block intracellular seeding (SH-SY5Y cells stably overexpressing mutant A53T human α-synuclein). Selected clones were sequenced and underwent an epitope mapping (PepperPrint assay). Finally, 306C7B3 was tested in the described *in vivo* Parkinson‘s Disease (PD) model in (Thy-1)-[A30P]-hα-synuclein mice.

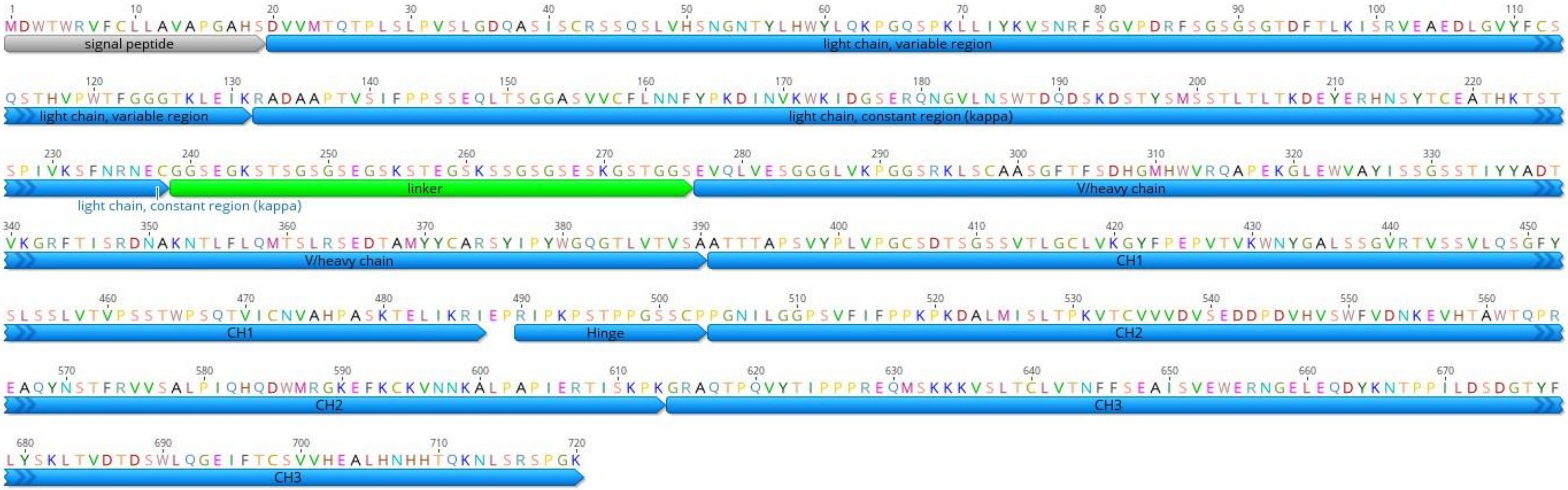

Supplementary Figure 2a. Amino acid sequence of murine scIgG-306C7B3.

Light and heavy chains are encoded within one single open reading frame due to the use of a linker sequence (green). The human IgG1 signal peptide was added to the primary antibody sequence to allow for efficient secretion of the scIgG from transduced cells (signal peptide).

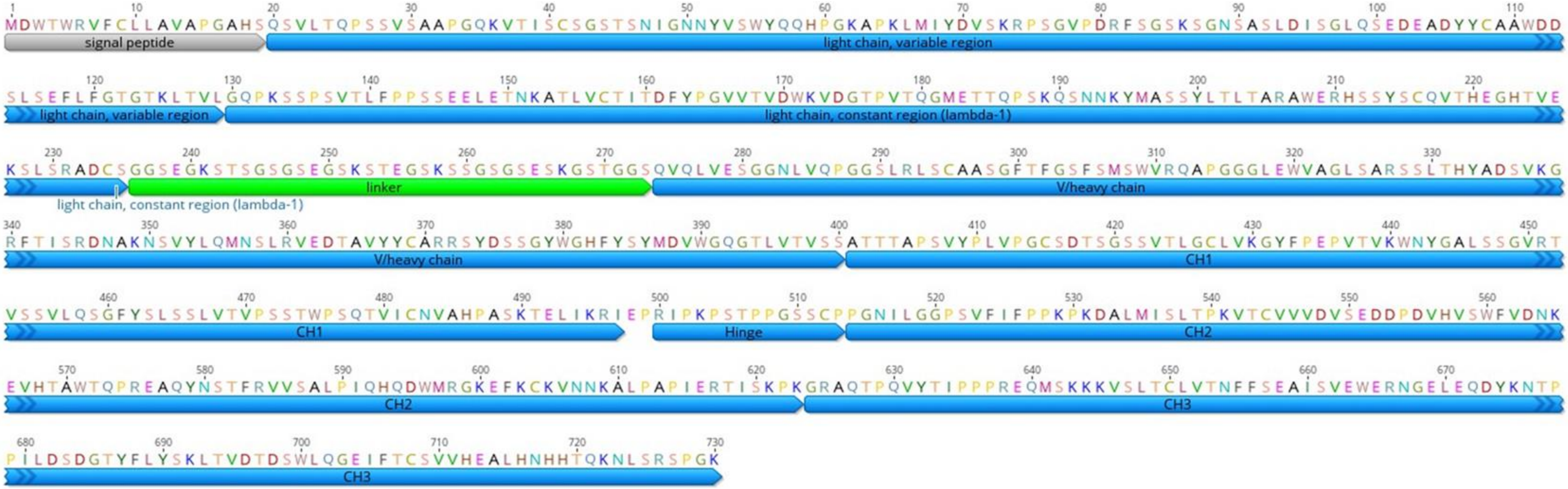

Supplementary Figure 2b. Amino acid sequence of murine scIgG-anti-FITC.

Light and heavy chains are encoded within one single open reading frame due to the use of a linker sequence (green). The human IgG1 signal peptide was added to the primary antibody sequence to allow for efficient secretion of the scIgG from transduced cells (signal peptide). The anti-FITC IgG sequence is derived from the scFv anti-FITC nanobody as described in Vaughan, T.J. et al. („Human antibodies with sub-nanomolar affinities isolated from a large non-immunized phage display library“. Nat. Biotechnol. 14, 309–314, 1996)

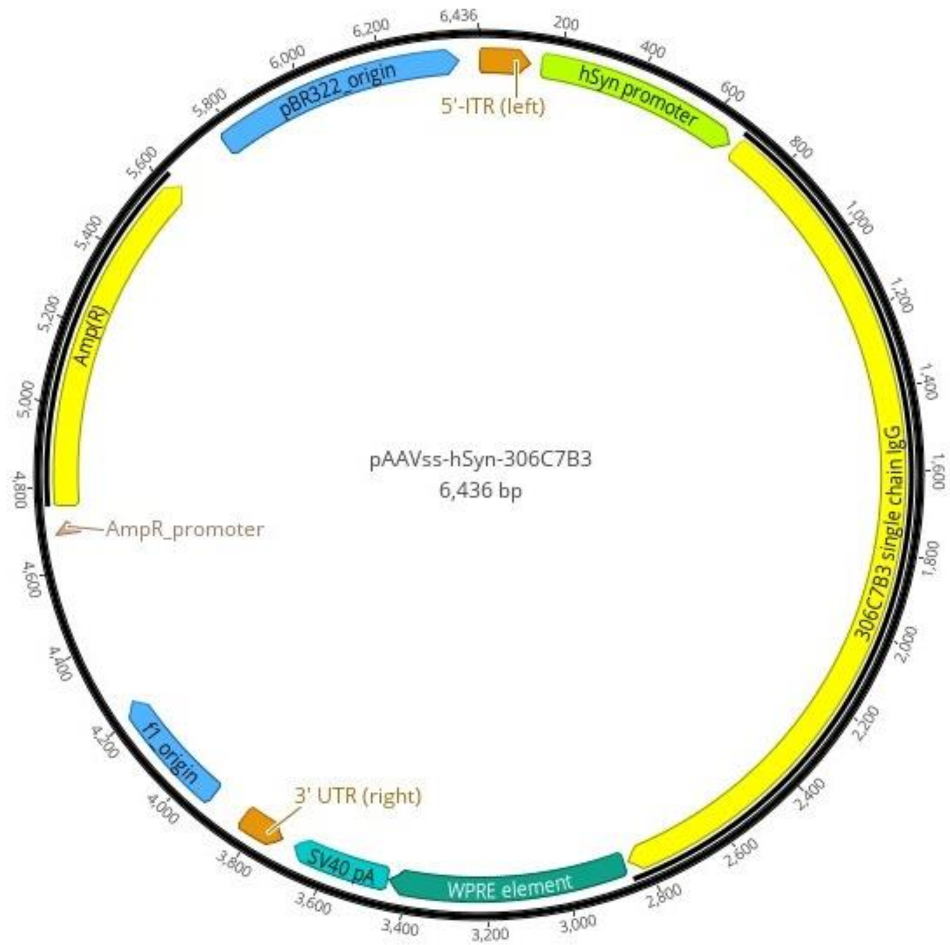

Supplementary Figure 2c. Plasmid map of pAAVss-hSyn-scIgG-306C7B3.
The expression cassette – flanked by the two inverted terminal repeats of AAV2 required for efficient packaging into AAV capsids – contains the human synapsin promoter (hSyn promoter), the full scIgG-306C7B3 antibody sequence in a single open reading frame including an optimized Kozak sequence, a WPRE element and the SV40 polyadenylation signal (SV40 pA).

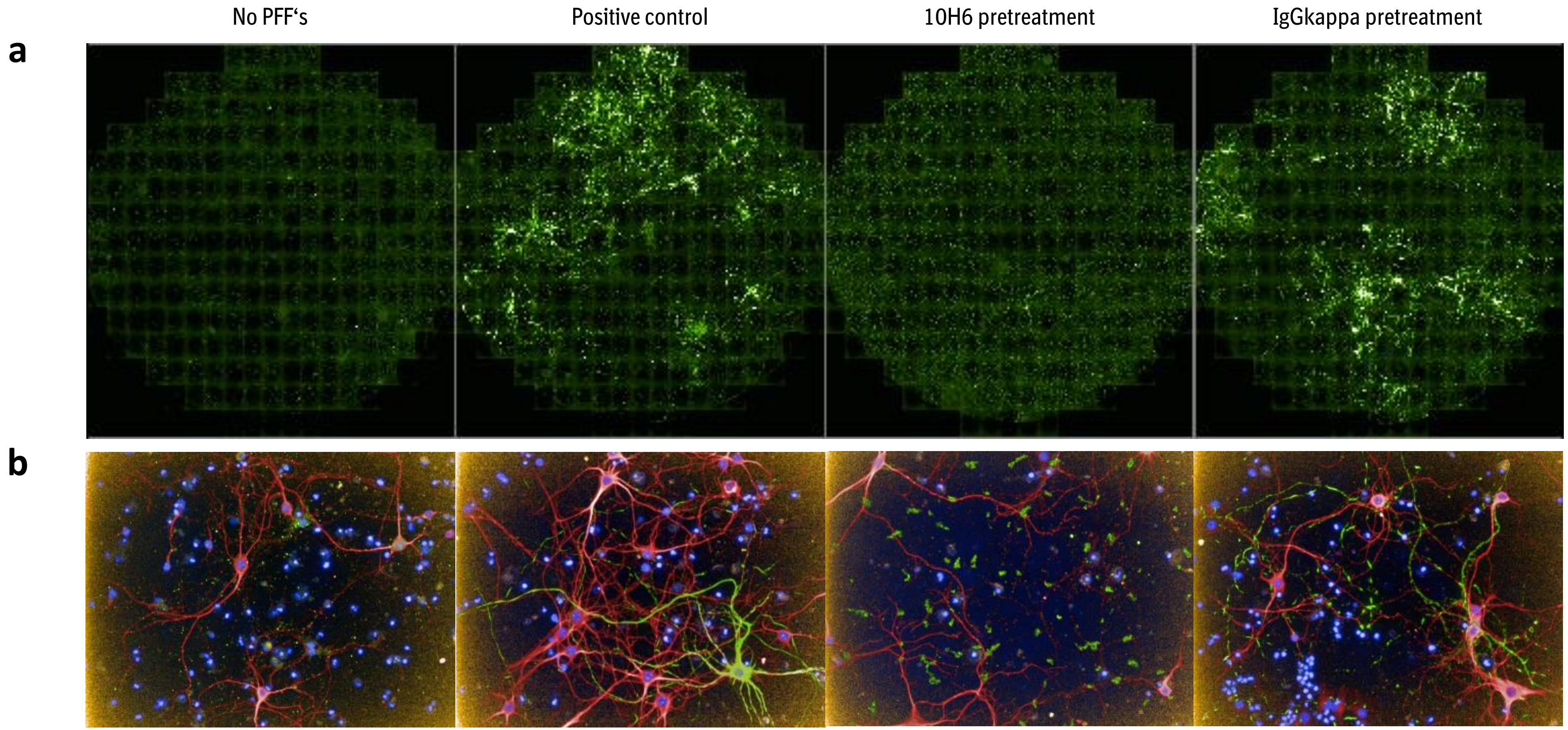

Supplementary Figure 3. ***In vitro*** **characterization of an aggregate specific α-synuclein antibody on primary cortical neurons.**

Primary cortical neurons (E16.5, 5.000 cells/well) from (Thy-1)-[A30P]-hα-synuclein mice overexpressing mutant A30P α-synuclein were incubated with 100ng of PFF human α-synuclein either untreated or pre-incubated with 1μg 10H6 prior to addition to the culture medium. 10H6 represents an aggregate-specific anti-human α-synuclein antibody similar to 306C7B3. After 21 days of *in vitro* culture, cells were fixed and stained for pSer129 α-synuclein (green) and MAP2 (red) as neuronal marker (DAPI nuclear staining in blue).

a, full plate view (96well microtiter plate) showing pSER129 α-synuclein stain only, indicating induced intracellular α-synuclein aggregation as marked by the pSer phosphorylation in the positive control as well as in primary neurons treated with a control antibody ("IgGkappa"). No intracellular pSer129 α-synuclein staining can be observed in 10H6 pretreated primary neurons. **b** Higher magnification showing individual neurons (marked by the red MAP2 staining). Note the presence of dot-like green immuno-fluorescence outside of neuronal cell bodies and axons, most likely due to a cross-reaction of the utilized secondary antibody for the pSer129 stain with the Fc-region of the 10H6 antibody bound to the human α-synuclein PFF's still present on the culture dish.

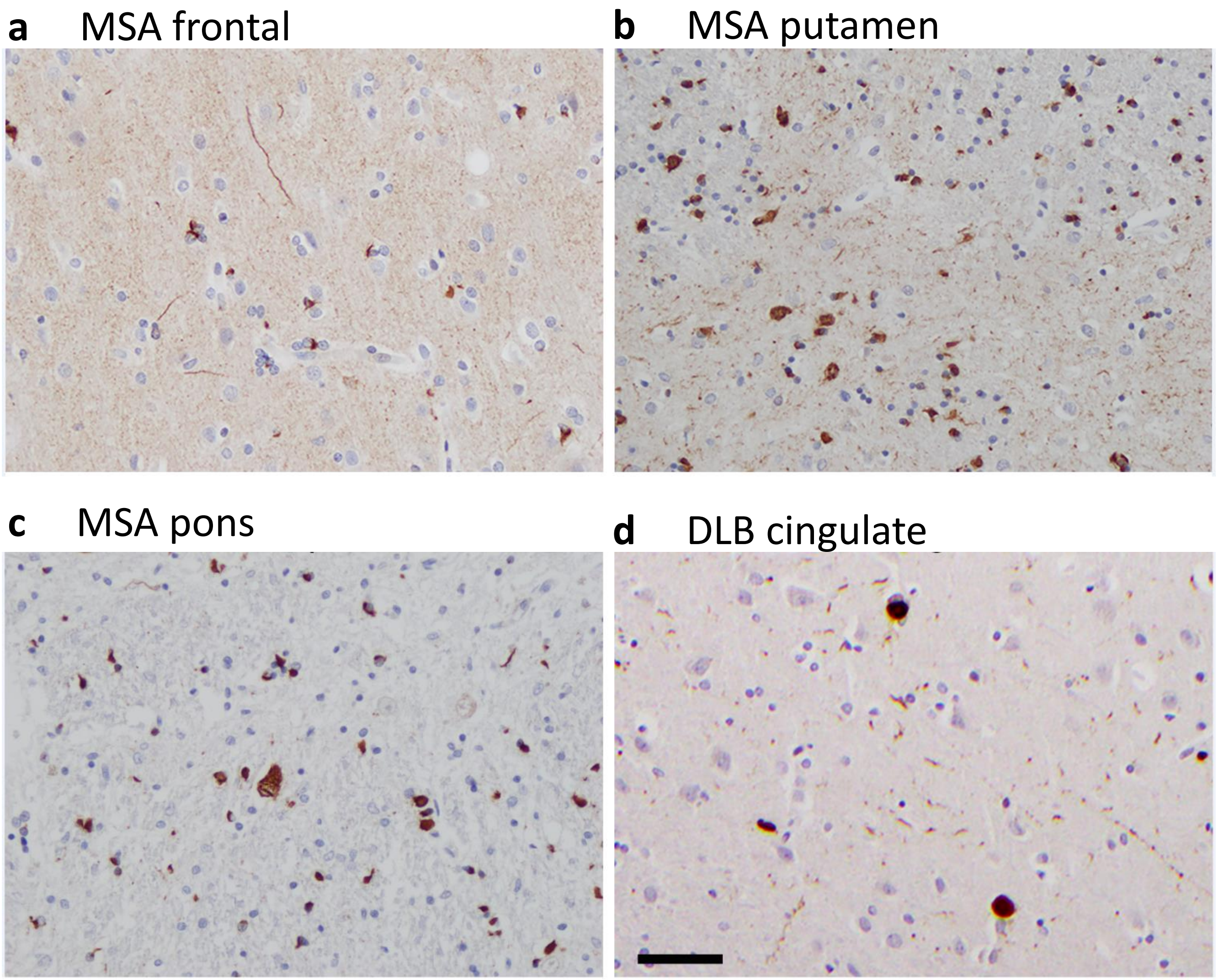

Supplementary Figure 4. 306C7B3 immunoreactivity in multiple system atrophy and Lewy body disease.

Immunohistochemistry for 306C7B3 in formalin-fixed paraffin embedded section of human postmortem tissue of α-synucleinopathies. Robust immunoreactivity is observed for all types of inclusions in MSA including cortical and subcortical neuronal and oligodendroglial inclusions in frontal cortex (**a**), putamen (**b**), and pons (**c**). In Lewy body diseases, strong labelling of Lewy bodies and Lewy neurites is seen as illustrated in cingulate gyrus of dementia with Lewy body case (**d**). Scale bar 50μm (a-d).

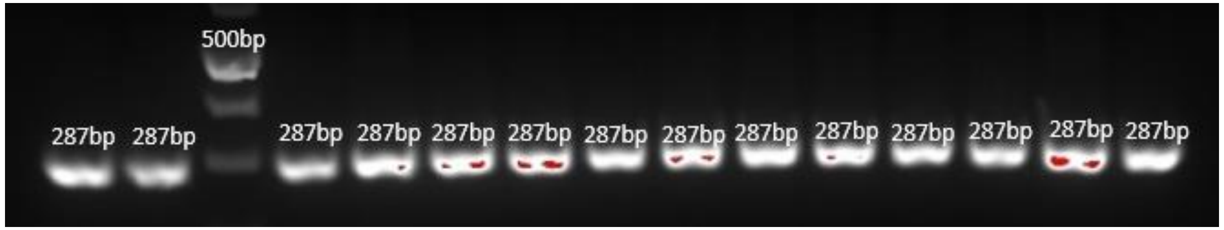

Supplementary Figure 5. Confirmatory biochemical analysis of all animals involved in the *in vivo* study.

Gel electrophoresis example showing the result of the genotype analysis via PCR. All animals in the study were shown to be homozygous for the A30P α-synuclein transgene as indicated by the amplified 287 bp band from genomic DNA isolated from each individual animal.

		Gender	loss of righting reflex	age at sacrifice	
Group	animal ID	(m / f)	(yes / no)	(days)	comments
control	8	m	yes	475	
	9	f	yes	605	
	10	f	yes	535	
	12	f	yes	574	
	13	m	no	466	found dead in cage
	14	m	no	551	large wound in the back and at the ear, euthanized
	15	m	yes	666	
	16	f	yes	522	
	17	f	yes	429	
	18	f	yes	597	
	19	f	yes	488	
	20	f	yes	590	
	111	f	yes	500	
	112	f	yes	548	
	172	m	yes	674	
	173	m	yes	612	
	174	m	yes	678	
	175	f	no	626	rectum prolapse, euthanized
	176	f	yes	554	
	177	f	no	575	found dead in cage
	178	f	no	443	found dead in cage
high dose AAV2HBKO-scIgG-antiFITC 2.0E+10 vg	1	f	yes	458	
	2	f	yes	468	
	3	f	yes	544	
	4	f	yes	593	
	5	m	yes	451	
	6	m	yes	617	
	7	m	yes	640	
	8	m	yes	493	
	143	f	yes	458	
	144	f	yes	665	
	146	m	yes	591	
	147	m	yes	540	
	168	m	yes	626	
	169	m	yes	674	
	170	m	yes	542	
	171	m	no	-	euthanized shortly after surgery
low dose	1	f	yes	576	
	2	f	yes	564	
	3	f	yes	591	

AAV2HBKO-scIgG-306C7B3 2.0E+09 vg	4	f	no	-	general deterioration shortly after surgery
	5	m	no	589	self inflicted large scale scratches, euthanized
	6	m	yes	607	
	7	m	yes	673	
	8	m	no	-	general deterioration shortly after surgery
	17	m	yes	603	
	18	m	yes	602	
	19	m	no	-	general deterioration shortly after surgery
	148	f	no	455	vaginal prolapse, euthanized
	149	f	no	-	general deterioration shortly after surgery
	150	m	yes	654	
	151	m	yes	644	
mid dose AAV2HBKO-scIgG-306C7B3 6.3E+09 vg	9	f	yes	566	
	10	f	yes	577	
	11	f	yes	613	
	12	f	yes	544	
	13	m	yes	508	
	14	m	yes	638	
	15	m	no	533	rectum prolapse, euthanized
	105	m	yes	669	
	152	f	yes	558	
	153	f	yes	634	
	154	m	yes	544	
	155	m	yes	586	
	164	f	yes	610	
	165	f	yes	557	
	166	m	no	-	general deterioration shortly after surgery
	167	m	yes	617	
high dose AAV2HBKO-scIgG-306C7B3 2.0E+10 vg	17	f	yes	538	
	18	f	yes	628	
	19	f	yes	564	
	20	f	yes	506	
	101	m	yes	660	
	102	m	yes	475	
	103	m	yes	736	
	104	m	no	572	rectum prolapse, euthanized
	139	f	no	566	large, focal swelling at tail, euthanized
	140	f	no	560	large weeping wound at left leg, euthanized
	141	m	yes	532	

	142	m	yes	588	
	159	m	yes	674	
	160	m	no	-	general deterioration shortly after surgery
	161	m	no	681	general deterioration, no loss of righting reflex
	162	m	yes	688	
	163	m	yes	625	

Supplementary Table 1.

Individual animal data for the *in vivo* functional study. Only animals with clear loss of righting reflex were included in the analysis. See comments for animals euthanized or found dead without loss of righting reflex.
